# *TB1*: from domestication gene to tool for many trades

**DOI:** 10.1093/jxb/eraa308

**Published:** 2020-08-06

**Authors:** Ernesto Igartua, Bruno Contreras-Moreira, Ana M Casas

**Affiliations:** 1 Estación Experimental de Aula Dei, EEAD-CSIC, Zaragoza, Spain; 2 European Molecular Biology Laboratory, European Bioinformatics Institute, Wellcome Genome Campus, Hinxton, UK

**Keywords:** Domestication genes, plant architecture, plant height, TCP transcription factors

## Abstract

This article comments on:

**Dixon LE, Pasquariello M, Boden SA**. 2020. TEOSINTE BRANCHED1 regulates height and stem internode length in bread wheat. Journal of Experimental Botany **71**, 4742–4750.

**Dixon *et al.* (2020) report a novel role for the *TB1* transcription factor gene in wheat, controlling plant height. This gene and its orthologues have long been known to affect branching in a number of crops and plant species. Its involvement in the determination of plant height opens up new avenues for the modification of this key trait, which affects multiple agronomic aspects of annual crops, from emergence to harvest index.**

## Plant height: key for modern agriculture

Plant height, the distance in centimetres from the ground to the tip of the spike (in wheat, for instance), is a deceptively simple trait with big agronomic repercussions. In the Poaceae family, it is affected by a number of signalling pathways, mainly (but not limited to) gibberellins (GAs), brassinosteroids, and strigolactones. This trait was at the core of the transformation of cereal cultivation spurred by the Green Revolution. Semi-dwarf varieties were an essential part of the genetic–technological package combining new lodging-resistant, high harvest index varieties (a high proportion of assimilates going to the grain), with the application of higher fertilizer rates. The widely known ‘*reduced height*’ alleles *Rht-B1* and *Rht-D1* were the protagonists of this story. They were the result of a ‘silver bullet’ approach, which reaped more profits than downsides. Briefly, germplasm was surveyed, and natural mutants with large effects on plant height were found and bred into adapted genetic backgrounds all over the world. This kind of approach still yields good results, as the study by Dixon *et al.* (2020) reveals. They report a novel effect of *TEOSINTE BRANCHED1* (*TB1*) on wheat height. However, the authors were not taking a shot in the dark. They targeted a domestication gene originally identified in maize, with known effects on plant architecture and fertility in a large number of species, including wheat, as demonstrated in a previous article by the same group (Dixon *et al.*, 2018). Exploring the function of domestication genes across species seems a sensible thing to do. These genes show different outcomes due to alternative modes of regulation, in many cases time and space dependent, rather than to simple differences in protein function (Dong *et al.*, 2019*a*). They are usually placed high up in gene hierarchy and, therefore, affect different pathways and, ultimately, a variety of phenotypic outputs. Domestication genes are the low-hanging fruits of crop genetics and, apparently, some are still waiting to be fully harvested.

## Genes that modulate plant height

The catalogue of major genes affecting plant height in wheat, without causing substantial deleterious agronomic effects, is not large. Even the genes broadly used in wheat breeding still present minor issues, as summarized in Dixon *et al.* (2020) and previous reports. Essentially, *Rht-B1* and *Rht-D1* present suboptimal seedling emergence, reduced biomass, and lowered fertility at temperatures above 24 °C. Given this last effect, it is not surprising that their presence in southern European cultivars is scarce (Würschum *et al.*, 2017). Other genes, such as *Rht18/24*, have been successfully used in breeding since well before their identification as quantitative trait loci (QTLs) (Würschum *et al* 2017). These genes, and the recently discovered *Rht25* (Mo *et al.*, 2018), all act in the GA signalling pathway. *Rht8* (Gasperini *et al.*, 2012) is the exception to this rule, as it responds to brassinosteroids, expanding the options for flexible tuning of plant height to breeding targets.

The discovery of new plant height genes is challenging, particularly for loss-of-function alleles, because forward genetics in wheat is complicated by the buffering effect of the homoeologue genes (Adamski *et al.*, 2020). The rich knowledge on Poaceae genes affecting plant height could be leveraged for wheat breeding, through either the search of natural variants, the induction of new alleles through gene editing, or the introduction of genes from wild relatives, barley, or rice. In fact, wheat breeding has exploited mainly the GA signalling pathway so far, while brassinosteroid effects are well known mostly in barley (Dockter and Hansson, 2015), with the mentioned exception of *Rht8*, and strigolactones in rice (Liu *et al.*, 2018).

The arrival of *TB1* is a welcome addition to the catalogue of wheat plant height genes, giving more leeway to breeders worldwide. Recently, Dixon *et al.* (2018) identified natural variation for *TB1* in bread wheat associated with changes in inflorescence architecture (see Box 1). In the present work, the same group goes one step further, indicating that *TB1* also regulates plant height, with the bonus of not affecting coleoptile length, and hence not compromising plant emergence. The *TB1* height-reducing effect, as described by Dixon *et al.* (2020), depends on gene dosage. The authors found a *highly branched* (*hb*) wheat line, characterized by reduced tillering and the formation of multiple paired spikelets in the inflorescence. The *hb* line is tetrasomic for chromosome 4D, containing two copies of *TB-D1*, and had higher expression of *TB1* in stems, which limited elongation. The role of *TB-D1* was indisputably validated through a transgenic approach. Additionally, the authors provide convincing confirmation of the involvement of another homoeologue, *TB-B1*, in plant height, via the comparison of two naturally occurring alleles. *TB-A1* was found to be weakly expressed and thus not considered. Natural alleles of wheat *TB1* genes are shown in Box 1, together with other TILLING alleles predicted to be deleterious in Ensembl Plants (Howe *et al.*, 2020), which could be interesting research materials to confirm the phenotypes observed by Dixon *et al.* (2020).

**Box 1. Natural and TILLING mutants of wheat *TB1* homoeologues**

Partial sequence alignment of the *TB-B1a* and *TB-D1a* alleles. Residue numbers correspond to *TB-B1a*. Natural variants found by Dixon *et al*. (2018, 2020) are indicated next to the SIFT score (bold) computed at Ensembl Plants. Two of them (D112Y and A271V) have scores <0.05 and are thus expected to be deleterious. Large dots mark other deleterious substitutions found in TILLING lines of wheat cultivars Kronos (tetraploid) and Cadenza (hexaploid), which can be browsed at Ensembl Plants (*TraesCS4B02G042700.1* and *TraesCS4D02G040100.1*) and ordered at SEEDSTOR. These and other resources for wheat are reviewed at Adamski *et al.* (2020). Secondary elements (yellow strands and pink helices), as well as the DNA-binding TCP domain (blue) and the R motif (green), are overlaid to give structural context to the mutants. For instance, in *Arabidopsis thaliana*, Davière *et al.* (2014) observed that DELLA proteins can interact with the TCP domain (just for class I TCP transcription factors), and mutants in that region affect plant height.

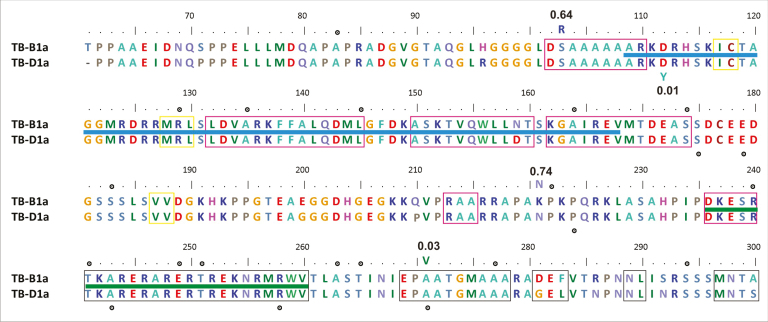

## *TB1*: ‘a finger in every pie’

It is surprising to realize the number of studies finding new roles for this gene in a large variety of species. The original *TB1* was discovered in maize, as the gene underpinning the shift from axillary branching to apical dominance that transformed teosinte into cultivated maize. Numerous orthologues found in other species such as Arabidopsis, barley, rice, or wheat, among many others, share its core functions of negatively regulating axillary bud outgrowth, and modulating inflorescence architecture (Doebley *et al.*, 1997; Takeda *et al.*, 2003; Aguilar-Martínez *et al.*, 2007; Ramsay *et al.*, 2011). *TB1* belongs to the Teosinte branched1/Cycloidea/Proliferating cell factor (TCP) class II gene family, a group of phylogenetically related, plant-specific transcription factors that share a non-canonical basic helix–loop–helix motif, the TCP domain (Nicolas and Cubas, 2016). Class II TCP genes are tightly regulated at multiple levels and prevent plant growth and proliferation. The function of the *TB1* gene is generally conserved across species, although cases which have several copies are common, and some may have adopted specialized functions (Box 2). Loss-of-function mutations of the gene are associated with increased branching or tillering. Increased dosage or overexpression results in reduced lateral branching, fewer tillers, and reduced culm length. Actually, *TB1* involvement in plant height had already been hinted at in wheat, and reported in rice (Choi *et al.*, 2012), as well as in maize (Studer *et al.*, 2017).

**Box 2. Number of *TB1* orthologues across vascular plants**

Dixon *et al.* (2018) discovered that the number of *TB1* genes in wheat is key to observe phenotypes. The bars are estimated *TB1* gene family sizes for several clades computed from a phylogenetic tree in Ensembl Plants. Note that subgenomes of polyploids were analysed on their own. The number of genomes in each clade is in parentheses. The maximum and minimum copies observed in each clade are also indicated, revealing that this gene family has systematically grown in some clades (Asteridae) or in individual species such as maize (8), soybean (10), or sunflower (16). There is evidence that some of these copies have neofunctionalized (Lyu *et al.*, 2020).

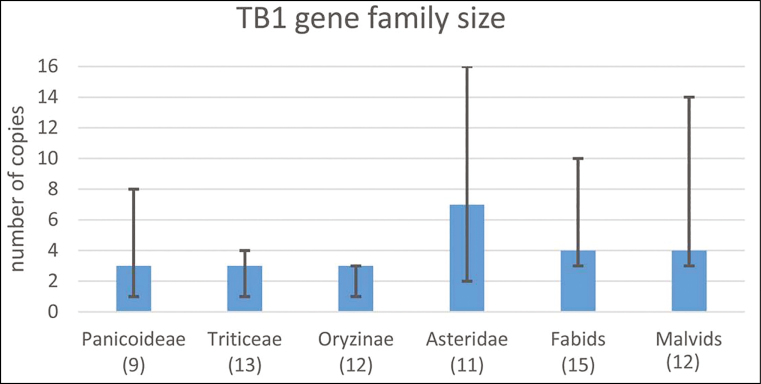

The mechanism of growth repression by *TB1* in grasses, or its orthologue gene *BRC1* in dicotyledonous species, is still not well known today. *TB1-like* genes seem to integrate signals from phytohormones (strigolactone, auxin, and cytokinins) and light stimuli (Nicolas and Cubas, 2016; Studer *et al.*, 2017) as part of the shade avoidance syndrome. Regulation of *TB1/BRC1* presents similarities and differences between monocots and dicots (Barbier *et al.*, 2019). In the latter group, *BRC1* is inhibited by increased sucrose availability and, in rice, it is targeted by *IPA1*, a functional transcription activator, with profound effects on rice architecture.

As a transcription factor gene, *TB1* targets other domestication loci in maize, including *teosinte glume architecture1* (*tga1*) and *prol1.1/grassy tillers1* (*gt1*), as well as its own promoter. It is also involved in regulating biosynthesis and downstream signalling of GAs, abscisic acid, and jasmonic acid (Dong *et al.*, 2019*b*). *TB1/BRC1* pathways are not fully elucidated in any species, but Dong *et al.* (2019*a*), in an extensive review, define *TB1* as ‘a master regulator operating in a large regulatory hierarchy that targets other domestication loci, all with wide phenotypic effects’. This view is confirmed in many studies which recurrently report that this gene and its orthologues are central integrators in multiple pathways. Among them, evidence in Arabidopsis and wheat demonstrates that *TB1* interacts with *FLOWERING LOCUS T1* (*FT1*), a gene well known to all crop scientists, which integrates signals from several flowering time pathways to induce the transition to reproductive growth. *TB1* modulates *FT1* activity in the axillary buds to prevent premature floral transition (Niwa *et al.*, 2013), reducing *FT1*-dependent activation of spikelet meristem identity genes (Dixon *et al.*, 2018), which affects spike fertility, hence yield potential. Given the dosage-dependent effect of *TB1* and its involvement in a wide diversity of physiological mechanisms, we hypothesize that the number of orthologues of this gene in plant species would provide some hints on the diversity of roles they might have adopted across vascular plants. Box 2 summarizes the number of orthologous copies of *TB1* on a variety of plant clades represented in Ensembl Plants.

## Further outlook

Future research efforts should aim at elucidating the interactions between genes affecting plant height in cereals. In this respect, the study of their gene regulatory networks, as proposed by Lavarenne *et al.* (2018), could shed light on their relationships, and point to potential synergies or redundancies that may guide their use, and even find other possible genes of interest. Additionally, wheat breeding would benefit from further research on pleiotropic effects of *TB1*, on genotype-by-environment interactions (are *TB1* effects dependent on temperature or water stress?), and genotype-by-management interactions (such as the response of *TB1* to widely applied growth regulators). The dynamics of expression of *TB1* underpin the multiple effects observed for this gene and its orthologues. For example, there is no report of *TB1* involvement in plant height in barley, but this is not surprising since its expression is detected only in inflorescences, and not in leaves or stems (Rapazote-Flores *et al.*, 2019). Also, *TB-A1* expression is almost absent in wheat stems (Dixon *et al.*, 2018). These results indicate that tissue-specific *TB1* regulation could be a sensible target for breeding in cereals.
